# Endovascular retrieval of a dislocated pushable coil in the common hepatic artery using a cerebral stent retriever

**DOI:** 10.1186/s42155-021-00224-8

**Published:** 2021-04-04

**Authors:** Mohammed Shamseldin, Albrecht Stier, Norbert Hosten, Ralf Puls

**Affiliations:** Radiology Department, Helios Klikum Erfurt, Nordhäuser Str. 74, 99089 Erfurt, Germany

**Keywords:** Upper gastrointestinal bleeding, Embolization, Coil dislocation, Stent retriever, pRESET, Foreign body removal

## Abstract

**Background:**

This is case of removing a dislocated pushable coil from the common hepatic artery (CHA) as a possible complication of using pushable coils in the embolization of an upper gastrointestinal bleeding (UGIB) from the gastroduodenal artery (GDA) by using a pRESET stent retriever (Phenox, Bochum, Germany) which is utilized mainly for treatment of endovascular stroke.

**Case presentation:**

An 88-year-old female patient was referred to our hospital to get an emergency embolization of the GDA causing an UGIB with a relevant drop of the hemoglobin level. During the routine embolization of the GDA using pushable coils, a complete dislocation of the last coil into the CHA took place leading to a relevant slowing down of the arterial blood flow to the liver. A decision was thereby made to remove the dislocated coil to avoid further possible complications which was successfully achieved.

**Conclusions:**

Various stent retrievers have been proven to be effective in removing dislocated coils during intracerebral coiling of different pathologies. This case report is to our knowledge the first case report proving the high efficacy and safety of using yet another stent retriever, namely a pRESET stent retriever in removing a fully dislocated coil in the abdominal vessels, namely in this case the CHA.

## Introduction

One of the most common and most serious cases facing an interventional radiologist during is an UGIB. The most common cause of an UGIB is a peptic ulcer disease from gastric and duodenal ulcers which is associated with high morbidity and mortality. Rapid endoscopic treatment to stop the bleeding is the treatment of choice. In about 15% of the patients, endoscopy is either unavailable or unsuccessful. In this case, endovascular therapy namely selective catheterization and embolization of the UGIB has emerged as an alternative to operative intervention in high-risk patients usually targeting the gastroduodenal artery which is the most source of UGIB due to its course behind the first part of the duodenum (Loffroy 2009). The gastroduodenal artery is a branch of the CHA which usually arises from the coeliac trunk.

Endovascular embolization of the GDA is however associated with rare complications such as coil dislocation. Inadvertent embolization of the CHA can result despite the dual blood supply of the liver to multiple complications, ranging from subtle elevation of the liver enzymes all the way up to hepatic infarction and even acute hepatic failure (Aina et al. 2001). Complication management including removal of dislocated coils is thereby essential. The effective use of stent retrievers, which are thrombectomy devices usually used in endovascular stroke treatment, was systematically investigated in animals (Nikoubashman et al. 2015a). On humans, this method has only been described in a few patients mainly with dislocated coils in intracranial vessels (Leslie-Mazwi et al. 2013; O'Hare et al. 2010; Liu et al. 2014; Hopf-Jensen et al. 2013). We present a case in which a fully detached coil was successfully extracted from the CHA using a cerebral pRESET stent retriever.

## Case report

**Fig. 1:**
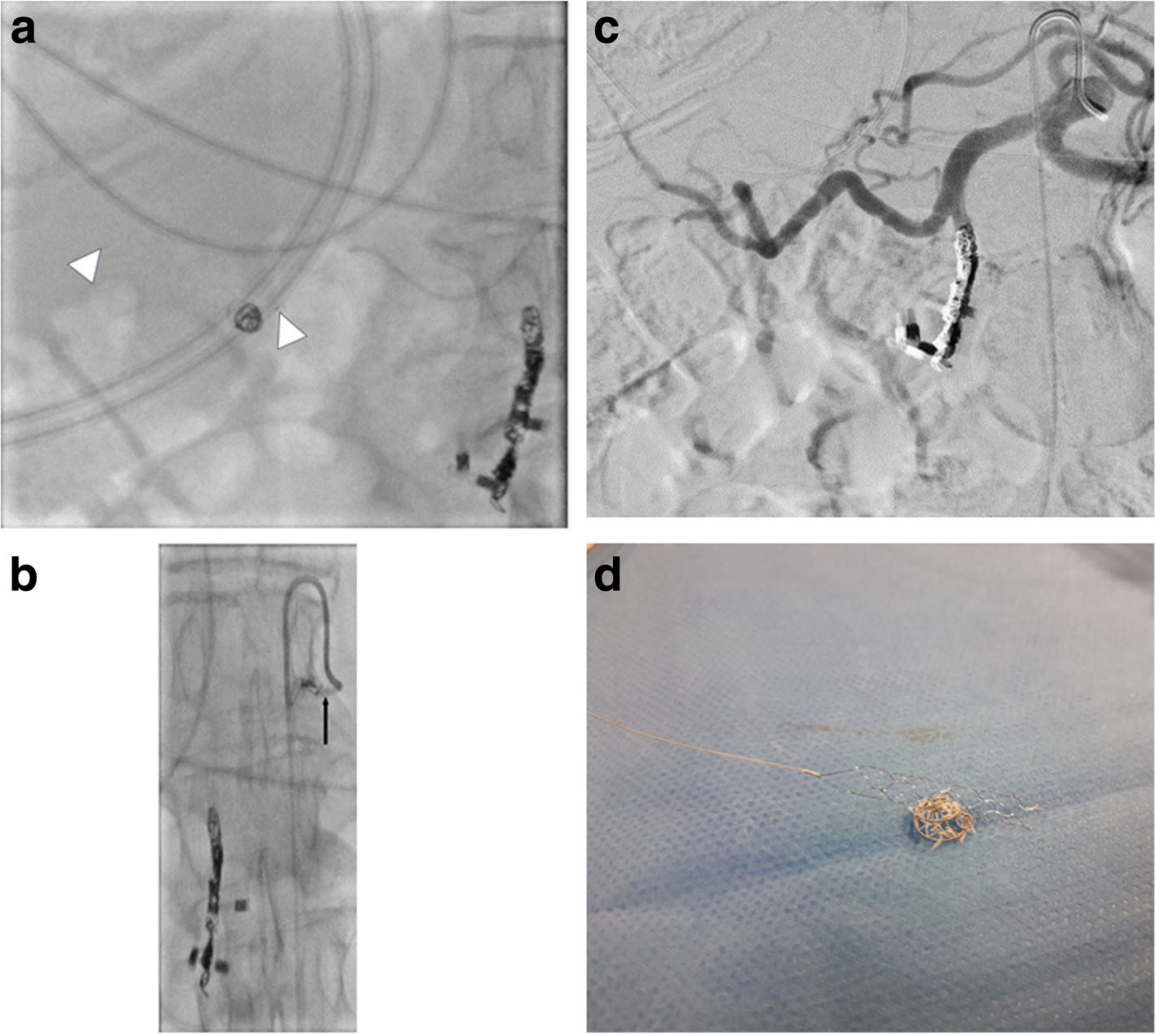
**a** pRESET stent retriever placed in the CHA covering the dislocated VortX-Diamond-18 6 × 6.7 mm pushable coil. Markers at both ends of the pRESET are visible (arrow heads) (**b**) advancement of the microcatheter (black arrow) and trapping the coil within the meshwork of the stent retriever by simultaneous pushing the microcatheter and pulling the pRESET. Coil removal by pulling the pRESET and microcatheter into the 5F SIM1-Catheter using gentle repetitive pulling movements (**c**) digital subtraction angiography documents regular flow after coil retrieval (**d**) removed dislocated coil hanging on the stent retriever

An elderly 88-year-old female patient suffering from an actively bleeding upper GIB due to a duodenal ulcer was treated with coil embolization of the GDA. Endovascular therapy was indicated after endoscopic management using three metal clips and the injection of 4 ml Suprarenin 1:100.000 failed to stop the bleeding. Embolization was carried out using pushable coils of various sizes in the usual “front door – back door” technique over a 2.7 French (F) Progreat microcatheter (Terumo, Tokyo, Japan) which is routinely used in our institute for such embolization procedures. During embolization, the last VortX-Diamond-18 pushable coil (Boston Scientific, Massachusetts, USA) measuring 6 × 6.7 mm was dislocated into the CHA causing a subsequent slowing down of the blood flow in the vessel. A decision was thereby made to remove the coil by retrieving it through the 5F SIM1-Catheter (Boston Scientific, Massachusetts, USA), which was already positioned in the coeliac trunk as a guiding catheter for the initial embolization of the GDA. An initial trial to remove the coil using a 4 mm Amplatz GOOSE-NECK® Microsnare Kit (ev3, Minneapolis, USA) was unsuccessful. Consequently, the decision was made to retrieve the dislocated coil using a cerebral pRESET stent retriever (4 × 20 mm). For this procedure, the initially used 2.7F Progreat microcatheter was brought distal to the dislocated coil and the pRESET was placed in the CHA covering the dislocated coil (Fig. 1a). The coil was easily pulled back all the way to the SIM1-Catheter. The first trial to retrieve the coil into the lumen of the SIM1-Catheter was however unsuccessful, with the coil dislocating back into the periphery of the CHA. In the second trial, an extra step to lock the dislocated coil within the meshwork of the pRESET was performed by carefully pushing the microcatheter to the position of the dislocated coil while pulling the stent retriever (Fig. 1b). The coil was then carefully retrieved by pulling back the pRESET together with the microcatheter into the SIM1-Catheter using gentle repetitive pulling movements. A postinterventional series showed a restoration of the normal flow without perforation or thrombosis of the CHA (Fig. 1c). The patient was symptom-free and stable with improvement of the Hemoglobin-level and with no changes in the liver enzymes.

## Discussion

Dislocation of coils during coil embolization is a quite rare complication but with possible vessel occlusion. The dislocation of coils occurs more frequently with usage of pushable coils in comparison to detachable coils due to the poor controllability. Dislocated coils occluding the blood flow in important vessels such as the CHA may be necessary to remove to avoid very rare but rather serious irreversible damage including acute hepatic failure (Aina et al. 2001). Recovery of dislocated coils with stent retrievers has been reported with various stent retrievers, including Solitaire (Medtronic, Minnesota, USA), Trevo (Stryker, Kalamazoo, MI) and Catch device (Balt, Montmorency, France). This method has proven to be highly effective in a few already published studies and case reports (Leslie-Mazwi et al. 2013; O'Hare et al. 2010; Liu et al. 2014; Hopf-Jensen et al. 2013; Kabbani et al. 2014).

The success rate in removal of dislocated coils in animals was over 99%, when trapping the coil between the stent retriever and the microcatheter was performed, regardless of the coil type, size and shape. In the same animal study, when the coil trapping maneuver was not applied, only 11% were successful (Nikoubashman et al. 2015a). This confirms our experience in this case where the coil was initially unsuccessful when the trapping maneuver was not used but efficiently removed as the microcatheter was advanced to trap the coil within the stent mesh of the pRESET. We do also recommend to unfold the stent retriever distally in the vessel with the dislocated coil being trapped at the most proximal part of the stent retriever or as to say the closest part of the stent retriever to the main catheter through which the coil would be removed.

While all published cases dealt with intracranial coils (Leslie-Mazwi et al. 2013; O'Hare et al. 2010; Liu et al. 2014; Hopf-Jensen et al. 2013; Kabbani et al. 2014) and only one case with a dislocated coil in the peroneal artery (Nikoubashman et al. 2015b), we have shown that retrieving dislocated coils in the abdominal vessels is also effective and this time using yet another available stent retriever (pRESET). To our knowledge is this case study the first one to deal with a coil dislocation in an abdominal vessel and the first one to use the pRESET stent retriever. In summary, endovascular coil recovery with stent retrievers can be considered an effective and safe treatment option in a wide range of retrieving foreign bodies in different body regions.

Another way to minimize the possibility of coils getting dislocated in the first place is to use detachable coils instead of pushable coils, due to the possibility in the former type to retract and reposition a dislocated coil as long as it is not detached from the pusher wire. The cost factor considering the expensive detachable coils might make this solution somewhat difficult to implement on a large scale.

## Conclusion

Coil dislocation is a rare complication during endovascular embolization of upper GIB, and in some cases essential to avoid irreversible ischemic damage. The recovery of dislocated coils with stent retrievers (in this case pRESET) is also effective in abdominal vessels especially if the trapping of the coil between the stent retriever and the microcatheter was achieved. Not using this locking maneuver significantly decreases the chances of a successful retrieval of the coil (Leslie-Mazwi et al. 2013; O'Hare et al. 2010; Nikoubashman et al. 2015a).

## Data Availability

“Not applicable.”
